# Supplementary material for: Phage-derived proteins and conjugates induce pilus detachment
Source: J Bacteriol. 2026 May 28;208(6):e00604-25. doi: 10.1128/jb.00604-25 (PMC13277300; doi:10.1128/jb.00604-25)
Supplement: Supplemental figures and tables — Figures S1 to S3, and Tables S1 and S2. [file jb.00604-25-s0001.pdf]

SUPPLEMENTAL INFORMATION

for

Phage-Derived Proteins and Conjugates Induce Pilus Detachment

Addison Frese, Zhi Zhao, Junjie Zhang, Lanying Zeng

List of content:

Supporting information text, including:

- Supplemental Tables S1 - S2
- Supplemental Figures S1 - S3

Supplemental Tables

Table S1: Strains, phages, or plasmids used in this study.

Bacterial Strain, Phage, or Plasmid	Description	Source
pET28-His-SUMO-Mat_{200}-sfGFP	Kan^R, truncated AP205 Mat protein with sfGFP fusion	Meng, R. et al. [1]
pET28-His-SUMO-Mat_{200}-AviTag	Kan^R, truncated AP205 Mat protein with an AviTag	This study
BirA expression plasmid	Cm^R, medium-copy expression plasmid of BirA utilized for biotinylation	Lab stock
A. higginsii	Host strain for AP205	ATCC, 17988
E. coli BL21 (DE3)	Protein expression strain	NEB, C2527H
AP205	ssRNA phage	Lab stock
Qβ	ssRNA phage	Lab stock
MS2	ssRNA phage	Lab stock
pET28-MBP-$Mat_{Qβ}$	Kan^R, the MBP tag linked with the full length of Qβ Mat protein	Reed et al.[2]
pZA32-MurAA	Kan^R, expression of MurAA to avoid cell lysis	Reed et al.[2]
pZE12-MS2coatA2V-sfGFP	Amp^R, MS2 coat protein linked with sfGFP to generate a fluorescent mosaic phage	Lab stock
MG1655	*E. coli* strain	Lab stock
HfrH	*E. coli* strain	Lab stock

Table S2: Primers used in this study.

Primer Name	Sequence	Source
pET_AviTag_F	atcttcgaggctcagaaaatcgaatggcacgaaTAACAAGCTTGCGGCCGCACTCGAG	This study
pET_AviTag_R	attttctgagcctcgaagatgtcgttcagaccGCTACCTCCGCCACCCGGGCTG	This study
pZA32_MurAA_F	TACTGAGCACATCAGCAGGA	This study
pZA32_MurAA_R	AGTTTCTGTAATCATGCTTG	This study
pZE12_F	TTTCGTCTTCACCTCGAGAA	This study
pZE12_R	TTTTATTTGATGCCTCTAGATTATTTGTAGAGCTC	This study
pET_Mat$_{Q\beta}$_F	AGGATTACGTTGTCGAATTCGACAATCTGTACCCT	This study
pET_Mat$_{Q\beta}$_R	AAGATACTATCAAGAACGTGTTTAAACGAACTAAAG	This study

Supplemental Figures

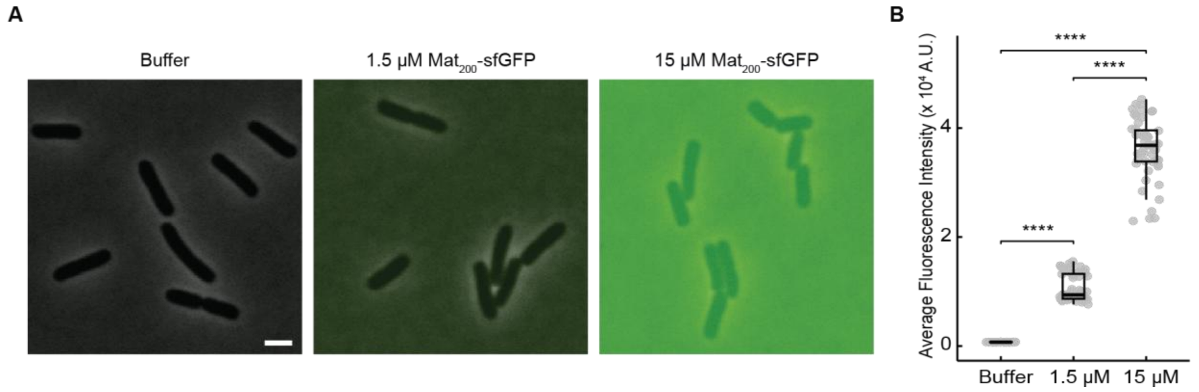

Figure S1: Mat200-sfGFP does not self-aggregate as long filaments in the absence of *A. higginsii* cells.

(A) Representative images illustrating *E. coli* MG1655 cells treated with either buffer or Mat200-sfGFP at concentrations of 1.5 μM or 15 μM. The LUTs for the GFP channel were adjusted from the minimum to the maximum settings to maximize signal visibility.

(B) The average fluorescence intensity of the entire frame increases significantly with Mat200-sfGFP concentration, reflecting the accumulation of unbound protein in the background rather than cell-specific binding. Grey dots indicate the intensity value of each individual frame measured. The total number of frames analyzed were 48 for buffer, 60 for 1.5 μM [Mat200-sfGFP], and 52 for 15 μM [Mat200-sfGFP]. **** indicates a p-value < 0.0001 (one-way ANOVA followed by Tukey's multiple comparison test). Experiments were performed with at least three biological replicates.

Scale bar, 2 μm.

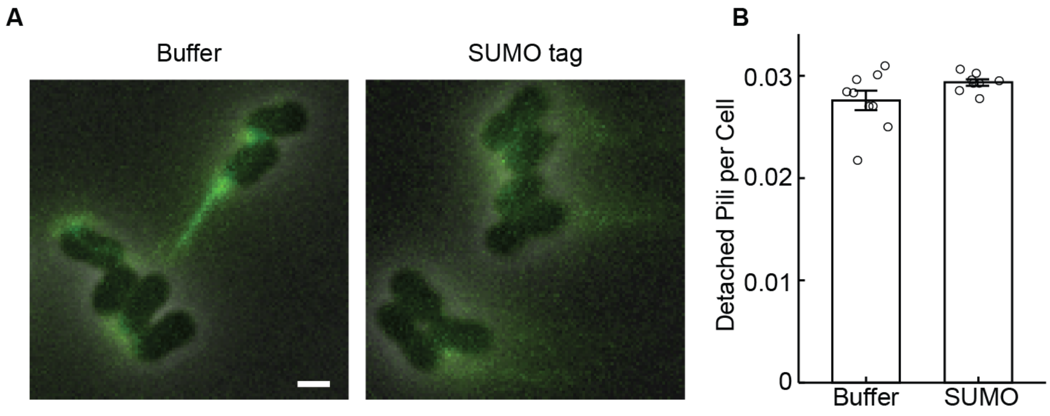

Figure S2: The SUMO tag alone does not detach T4P of *A. higginsii*.

(A) Representative images treated with the SUMO tag alone or buffer with the T4P illuminated with Mat_{200}-sfGFP.

(B) The number of detached pili per cell remains negligible for the SUMO tag control compared to buffer treatment. The total number of detached pili per cell were 116/3908 for the SUMO tag and 131/4635 for the buffer control. Detached pili per cell were quantified by dividing the total number of detached pili by the total number of cells. White dots represent individual technical replicates from at least three biological replicates.

Scale bar, 2 µm. All error bars in this figure represent SEM.

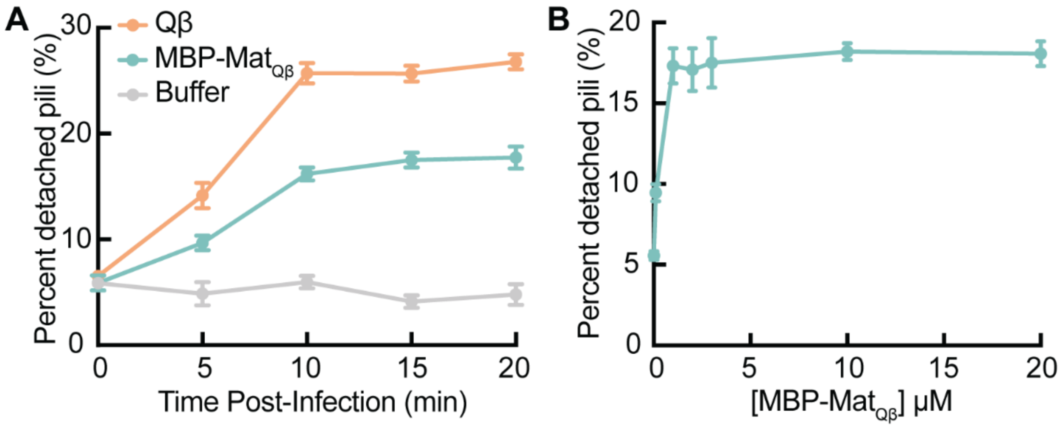

Figure S3: MBP-Mat$_{Qβ}$ detaches F-pilus.

(A) The percent of detached pili was quantified at different time points (0, 5, 10, 15, and 20 min) post-infection/treatment for Qβ, MBP-Mat$_{Qβ,}$ and buffer. The number of detached pili per total pili quantified post-infection for Qβ was 80/1306, 182/1197, 330/1266, 296/1131, and 306/1120, respectively; for MBP-Mat$_{Qβ}$, 62/1199, 113/1237, 192/1214, 218/1293, and 2091263, respectively; and for buffer, 69/1198, 77/1304, 66/1182, 48/1049, and 63/1104, respectively. Every experiment was performed with at least three biological replicates.

(B) The percent of detached pili was quantified after 10 min of treatment with different concentrations of MBP-Mat$_{Qβ}$ (0, 0.1, 1, 2, 3, 10, and 20 μM). The number of detached pili per total pili quantified for the MBP-Mat$_{Qβ}$ concentrations was 71/1295, 125/1336, 210/1217, 219/1292, 246/1410, 201/1105, and 211/1175. Each concentration was tested with at least three biological replicates.

Percent detached pili (%) was quantified by diving the total number of detached F-pili by the total number of labeled F-pili and then multiplied by 100. All error bars in this figure represent SEM.

SI References

1. Meng R, Xing Z, Chang JY, et al. Structural basis of Acinetobacter type IV pili targeting by an RNA virus. *Nat Commun*. 2024;15(1):2746. doi:10.1038/s41467-024-47119-5

2. Reed CA, Langlais C, Kuznetsov V, Young R. Inhibitory mechanism of the Qβ lysis protein A2. *Molecular Microbiology*. 2012;86(4):836-844. doi:10.1111/mmi.12021
